# Expert Opinions on the Most Promising Treatments and Vaccine Candidates for COVID-19: Global Cross-sectional Survey of Virus Researchers in the Early Months of the Pandemic

**DOI:** 10.2196/22483

**Published:** 2021-02-26

**Authors:** Bernardo Pereira Cabral, Luiza Braga, Fabio Mota

**Affiliations:** 1 Faculty of Economics Federal University of Bahia Salvador Brazil; 2 Center for Strategic Studies Oswaldo Cruz Foundation Rio de Janeiro Brazil; 3 Faculty of Economics Fluminense Federal University Niterói Brazil

**Keywords:** COVID-19, SARS-CoV-2, vaccine, treatment, survey, public health, drug, clinical trial

## Abstract

**Background:**

The COVID-19 pandemic presents a great public health challenge worldwide, especially given the urgent need to identify effective drugs and develop a vaccine in a short period of time. Globally, several drugs and vaccine candidates are in clinical trials. However, because these drugs and vaccines are still being tested, there is still no definition of which ones will succeed.

**Objective:**

This study aimed to assess the opinions of over 1000 virus researchers with knowledge on the prevention and treatment of coronavirus-related human diseases to determine the most promising drug and vaccine candidates to address COVID-19.

**Methods:**

We mapped the clinical trials related to COVID-19 registered at ClinicalTrials.gov. These data were used to prepare a survey questionnaire about treatments and vaccine candidates for COVID-19. In May 2020, a global survey was conducted with authors of recent scientific publications indexed in the Web of Science Core Collection related to viruses, severe acute respiratory syndrome coronavirus, coronaviruses, and COVID-19.

**Results:**

Remdesivir, immunoglobulin from cured patients, and plasma were considered to be the most promising treatments in May 2020, while ChAdOx1 and mRNA-1273 were considered to be the most promising vaccine candidates. Almost two-thirds of the respondents (766/1219, 62.8%) believed that vaccines for COVID-19 were likely to be available in the next 18 months. Slightly fewer than 25% (289/1219, 23.7%) believed that a vaccine was feasible, but probably not within 18 months.

**Conclusions:**

The issues addressed in this study are constantly evolving; therefore, the current state of knowledge has changed since the survey was conducted. However, for several months after the survey, the respondents’ expectations were in line with recent results related to treatments and vaccine candidates for COVID-19.

## Introduction

In December 2019, in Wuhan, China, a new coronavirus disease characterized by fever, cough, dyspnea, chills, muscle pain, sore throat, and loss of taste and smell [1] rapidly spread worldwide and was declared a pandemic by the World Health Organization (WHO) on March 11, 2020 [2]. By September 1, 2020, this novel coronavirus disease, COVID-19, caused by SARS-CoV-2, had resulted in more than 860,000 deaths in 216 countries, areas, or territories [3]. According to the WHO, the focus of disease management efforts to date has been on infection prevention, detecting and monitoring cases, and supportive care [4]. Currently, there is no specific treatment for COVID-19; instead, the disease is managed using a large group of experimental therapies of different types [5]. Currently, hundreds of registered clinical trials worldwide are testing the dosages and adequacy of different drugs to treat different stages of the disease [6]. Similar efforts are being made to create a vaccine [7,8]. Finding the most promising lines of treatment and vaccines to reduce the burden of COVID-19 is probably the greatest scientific and technological challenge of our time.

Despite the myriad of therapeutics and vaccine candidates being tested worldwide, it is still unclear which ones will prove successful in addressing COVID-19. Some previous studies have attempted to map the most promising treatments and vaccine candidates to fight COVID-19 [6-8]; however, these studies place little or no emphasis on experts’ opinions. Our study addresses this gap by assessing the opinions of over 1000 experts involved in virus research worldwide with knowledge on the prevention and treatment of coronavirus-related human diseases. Clinical trial data were obtained from ClinicalTrials.gov, and the survey’s respondents were identified from scientific publications indexed in the Web of Science (WoS) Core Collection related to viruses, severe acute respiratory syndrome coronavirus (SARS-CoV), coronaviruses, and COVID-19.

## Methods

We mapped the clinical trials related to COVID-19 registered at ClinicalTrials.gov (Multimedia Appendix 1) using the Aggregate Analysis of ClinicalTrials.gov (AACT) database, maintained by the Clinical Trials Transformation Initiative [9]. The search was conducted on April 23, 2020, via the PostgreSQL AACT connection. It retrieved 406 clinical trials with coronavirus as the variable condition (diseases being tested). The records were imported into VantagePoint, version 11.0 (Search Technology Inc); we then selected only the trials related to COVID-19 by searching the condition, title, outcome measure, keywords, and Medical Subject Headings (MeSH) conditions fields for the following descriptors: *Covid-19*, *Covid19*, *Covid 19*, *Coronavirus-19*, *Coronavirus 19*, *Coronavirus 2019*, *Sars-CoV2*, *SARS-CoV-2*, *SARS COV 2*, *Novel Coronavirus*, *2019 Novel Coronavirus*, *2019-Novel coronavirus*, *2019-nCoV*, *2019nCoV*, and *Coronavirus Disease 2019*.

This search retrieved 361 clinical trials: 237 (65.7%) were interventional studies, in which biomedical interventions were assigned and tested, and 124 (34.3%) were observational studies, in which no specific intervention was assigned by the principal investigator. Considering the purpose of this study, observational studies were not included in the analysis. Of the 237 interventional studies, only those pertaining to drugs (n=173, 73.0%) and biologicals or vaccines (n=31, 17.9%) were selected. Clinical trials whose status was withdrawn (Phase 2) or suspended (Phase 1) were excluded. To select only clinical trials of vaccine candidates, we searched the studies of biologicals and vaccines for the terms *vaccine* and *vaccines* in the following fields: interventions, keywords, MeSH interventions, outcome measures, study designs, and title. At the end of these procedures, 170 clinical trials with drug interventions and 6 clinical trials with vaccine interventions were selected for analysis.

Then, these clinical trial data were used to prepare the survey questionnaire, which was structured in four parts. The first part was an introduction to the survey (purpose, respondents’ role, estimated time to complete, time of availability of the questionnaire, confidentially and privacy, and consent for the use of data provided). The second part asked about the respondents’ knowledge level regarding the prevention and treatment of coronavirus-related human diseases. Respondents who reported having no knowledge of these topics were disqualified from the survey and did not answer the remaining questions. The third part covered the vaccine candidates and therapeutics currently in clinical trials and required the respondents to choose the ones they believed were most likely to be successful in treating or preventing COVID-19.

The fourth part of the survey consisted of demographic questions. The results of this study were not influenced by the demographics of the respondents, which were assumed to be relatively homogeneous because the respondents were all authors of scientific publications related to viruses, coronaviruses, SARS-CoV, and COVID-19, with knowledge on prevention and treatment of coronavirus-related human diseases. Nevertheless, we asked a few demographic questions to provide a broad overview of the respondents’ profiles. These questions were placed at the end of the questionnaire to prevent fatigue from answering the main questions, which could increase the number of skipped questions and the overall dropout rate.

The respondents to this survey were authors of recent scientific publications indexed in the WoS related to viruses, coronaviruses, SARS-CoV, and COVID-19. These publications were identified using the query shown in Textbox 1.

**Query used to identify the publications by respondents to the survey.**

ti=(virus*) OR ts=(Coronavirus* OR Alphacoronavirus* OR Betacoronavirus* OR Deltacoronavirus* OR Gammacoronavirus* OR “SARS Virus*” OR “Severe Acute Respiratory Syndrome Virus*” OR “SARS-Related Coronavirus*” OR “SARS-CoV” OR “SARS-Associated Coronavirus*” OR “SARS Coronavirus*” OR “Severe acute respiratory syndrome-related coronavirus*”) OR ts=(“COVID-19” OR COVID19 OR “2019 novel coronavirus” OR “SARS-CoV-2” OR “2019-nCoV” OR “coronavirus disease 2019” OR “coronavirus disease-19”)

Refined by: research areas (Virology OR Infectious Diseases OR Immunology OR Research Experimental Medicine OR Pharmacology Pharmacy OR General Internal Medicine)

Indexes=SCI-EXPANDED Timespan=2017-2020

The query searched all types of documents published from 2017 to May 2020 with the term *virus* in their titles or terms related to SARS-CoV, coronavirus, or COVID-19 in their title, abstract or keywords. The terms used in this query were collected in the MeSH index of the US National Library of Medicine [10], which is a controlled and hierarchically organized vocabulary used for indexing articles for PubMed [11]. We used only the Science Citation Index Expanded (SCI-EXPANDED) to identify respondents with publications in science journals. We then restricted the results to articles published in research areas related to virology, infectious diseases, immunology, experimental medicine, pharmacology, pharmacy, and general internal medicine.

The search returned 21,486 records of publications, which were imported into the data/text mining software VantagePoint 11.0 to generate a comma-separated values (CSV) file of the authors’ information (name, email, and publication title). From the information gathered, 23,428 unique emails were identified. Using an in-house Python script, we then linked approximately 84% of these emails to their account owners, which enabled personalized emails to be sent to most of the respondents. All 23,428 emails were uploaded to SurveyMonkey, the web-based survey platform that was used to build the questionnaire and to manage and conduct the web-based survey. After uploading, the number of emails was reduced to 22,879 due to previously opted-out contacts in SurveyMonkey and bounced emails.

Before the formal survey, the questionnaire was validated in a pilot study with a random sample of 10% of the 3585 emails (after uploading to SurveyMonkey) that were not linked to their account owners (unlinked emails). It is known that personalized invitation emails positively influence the response rates of web-based surveys [12]. Therefore, due to the need to obtain a substantial number of responses to be able to report statistically meaningful results, we opted to use only unlinked emails in the pilot. The results and feedback from some of the 20 respondents who took part in this pilot were used to make some changes in the original questionnaire; therefore, the data collected in the pilot were not included in the survey’s results.

**Figure 1:**
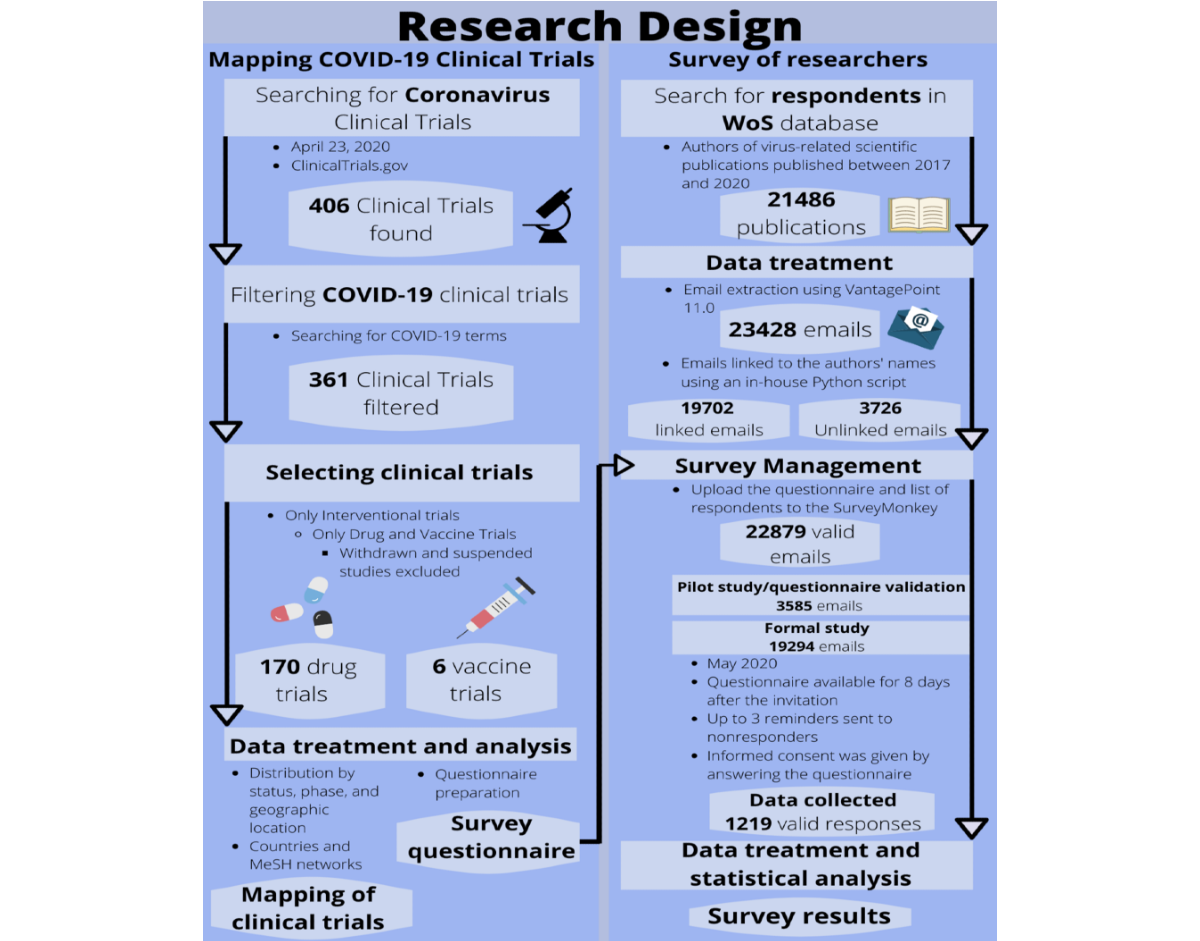
Research design used in the study. MeSH: Medical Subject Headings; WoS: Web of Science.

The pilot and the formal survey were conducted in May 2020. The invitation and reminder emails provided information about the study that was similar to that in the first part of the questionnaire itself (described above). The web-based questionnaire was available for completion on SurveyMonkey for 8 days after the email invitation was sent, and up to three reminder emails were sent to nonresponders. Figure 1 summarizes the research design.

## Results

A total of 1275 researchers agreed to participate in this study; however, 45 (3.5%) reported having no knowledge of the prevention or treatment of coronavirus-related human diseases and were disqualified from the survey, leaving 1230 valid responses. Of these respondents, 59.8% (735/1230) reported having good knowledge of the subject, and 40.2% (495/1230) said they had some knowledge. The sample of completed questionnaires (1050/1230, 85.4%) can be considered representative, with a 95% confidence level and a 3% margin of error. To ensure clarity in the graphic representation and description of the results, we combined the answers of these two groups of respondents. Statistically significant differences between them are reported in the descriptions of the results when pertinent. As the questions on therapeutics and vaccine candidates were mandatory in the survey, we gave the respondents an “I prefer not to answer” option. However, because these answers may reflect neutral opinions, we chose not to include them in the analysis of the results. The results by the respondents’ level of knowledge, including for the answer “I prefer not to answer,” are available in Multimedia Appendix 2.

**Figure 2:**
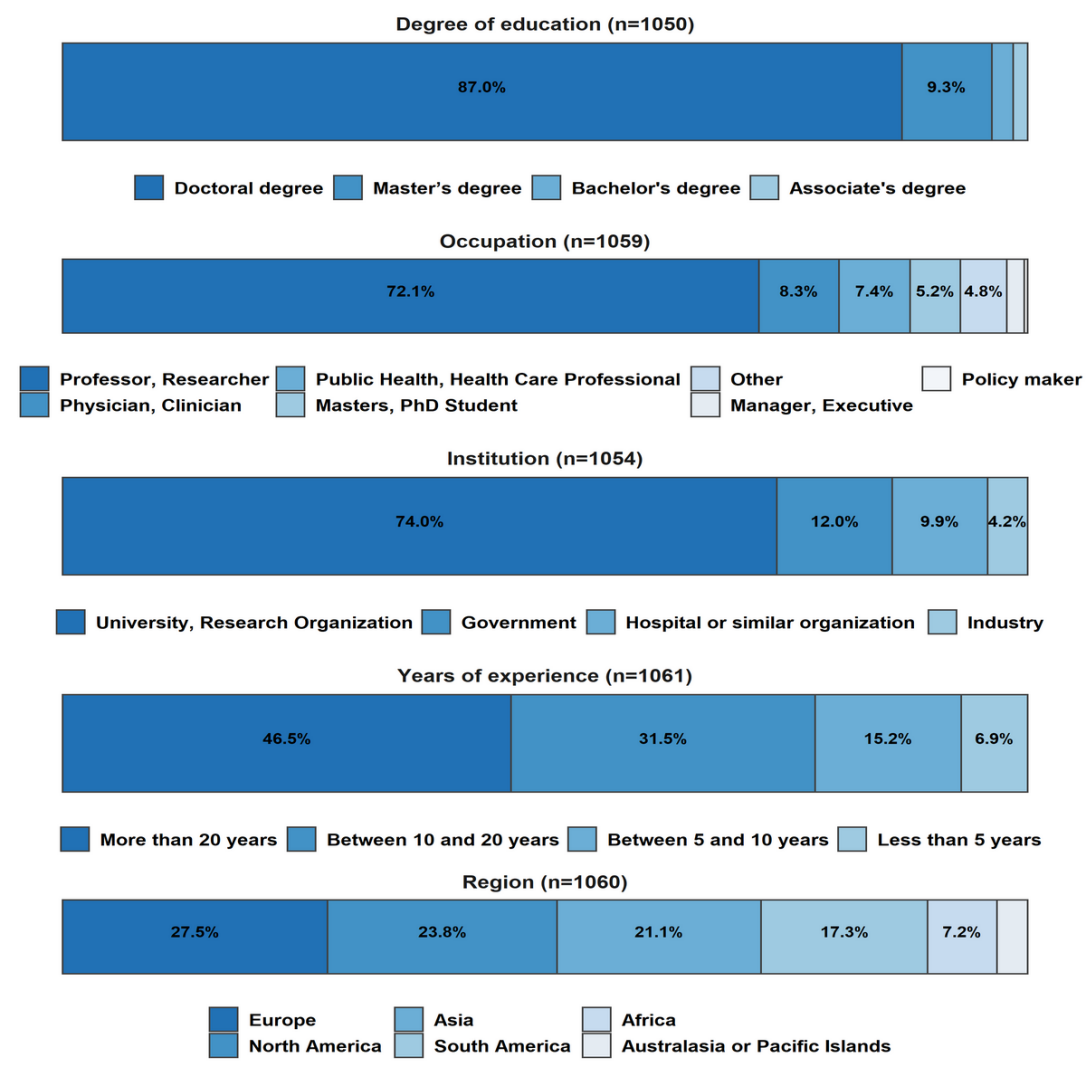
Demographic overview of the survey respondents.

Figure 2 shows the demographic characteristics of the respondents. Most of them (913/1050, 87.0%) held a doctoral degree, and 9.3% (98/1050) held a master’s degree. The majority of the respondents were professors or researchers (764/1059, 72.1%), followed by physicians or clinicians (88/1059, 8.3%) and public health or health care professionals (78/1059, 7.4%). Of the respondents, 74% (780/1054) worked at universities or research organizations, 12.0% (126/1054) in government, and 9.9% (104/1054) at hospitals or similar organizations. A few respondents worked in industry (44/1054, 4.2%). Approximately 50% (493/1061, 46,5%) had more than 20 years of experience in their fields of expertise, while slightly over 30% (334/1061, 31,5%) had 10 to 20 years of practice. The respondents were similarly distributed across Europe (291/1060, 27.5%), North America (252/1060, 23.8%), Asia (224/1060, 21.1%), and South America (183/1060, 17.3%). Much smaller numbers of respondents came from Africa and Australasia and the Pacific Islands: 7.2% (76/1060) and 3.2% (34/1060), respectively.

**Figure 3:**
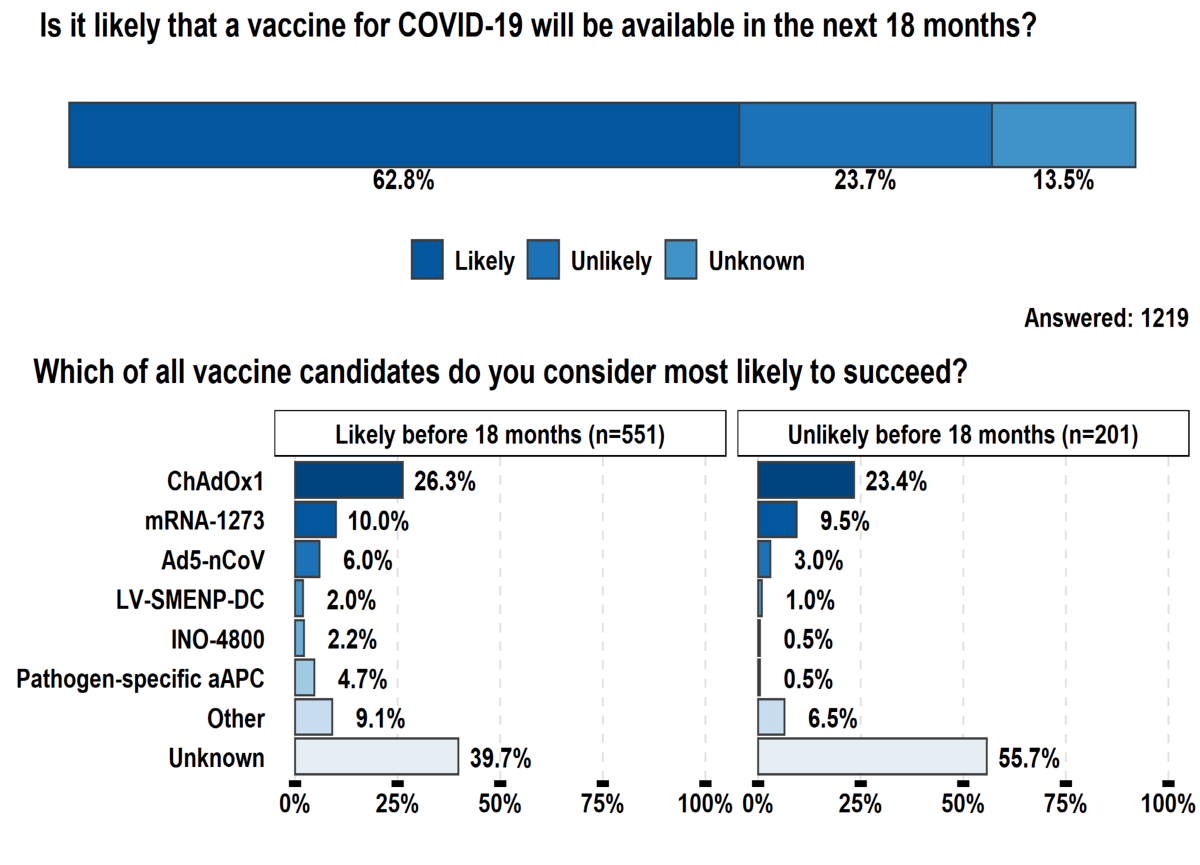
Survey respondents’ expectations regarding whether a vaccine for COVID-19 will be available in the next 18 months, and their choices of the most promising vaccine candidates.

The respondents were asked to share their opinion on whether a vaccine for COVID-19 would be available in the following 18 months. Considering that the questionnaire was administered in May 2020, this deadline was October 2021. They were given three choices: the first two were “likely” and “unlikely,” which led them to a question about vaccine candidates, and the third was “unknown,” which led them directly to the questions related to the treatment of COVID-19 and skipped the questions about vaccine candidates. Figure 3 shows the results. Almost two-thirds of the respondents (766/1219, 62.8%) believed that vaccines for COVID-19 were likely to be available in the next 18 months. Slightly fewer than 25% (289/1219, 23.7%) believed that a vaccine was feasible, but probably not within 18 months. A small minority (164/1219, 13.5%) did not know whether a vaccine would be available within 18 months. As we now know, several countries started administering vaccinations in December 2020, almost one year ahead of our suggested deadline.

On April 23, 2020, there were 6 vaccine candidates for COVID-19 in phase 1 or phase 2 clinical trials according to data collected from ClinicalTrials.gov. Accordingly, these were the only candidates offered as options for the respondents (Figure 3). The respondents who believed the development of a successful vaccine was likely, whether in the next 18 months or not, believed ChAdOx1, from the University of Oxford, to be the most promising candidate, followed by the mRNA-1273 vaccine, from Moderna. Breaking down the responses into the two groups, ChAdOx1 and mRNA-1273 were the top candidates, respectively, for 26.3% (n=145) and 10.0% (n=55) of the 551 respondents who believed that a vaccine would be available within 18 months, and 23.4% (n=47) and 9.5% (n=19) of the 201 respondents who believed that more time was needed. The respondents with self-reported good knowledge of the subject, irrespective of the time they thought the vaccine would take to be developed, had a significantly higher preference for ChAdOx1 than those with some knowledge (*P*=.05). Figure 3 also shows that many of the respondents reported that it was still unknown which vaccine candidate was the most likely to succeed—39.7% (219/551) of respondents who believed a vaccine was likely to be available before 18 months and 55.7% (112/201) of respondents who believed a vaccine was unlikely to be available before 18 months. A much smaller number considered that none of the 6 vaccine candidates were the most promising—9.1% (50/551) of respondents who believed a vaccine was likely to be available before 18 months and 6.5% (13/201) of respondents who believed a vaccine was unlikely to be available before 18 months.

**Figure 4:**
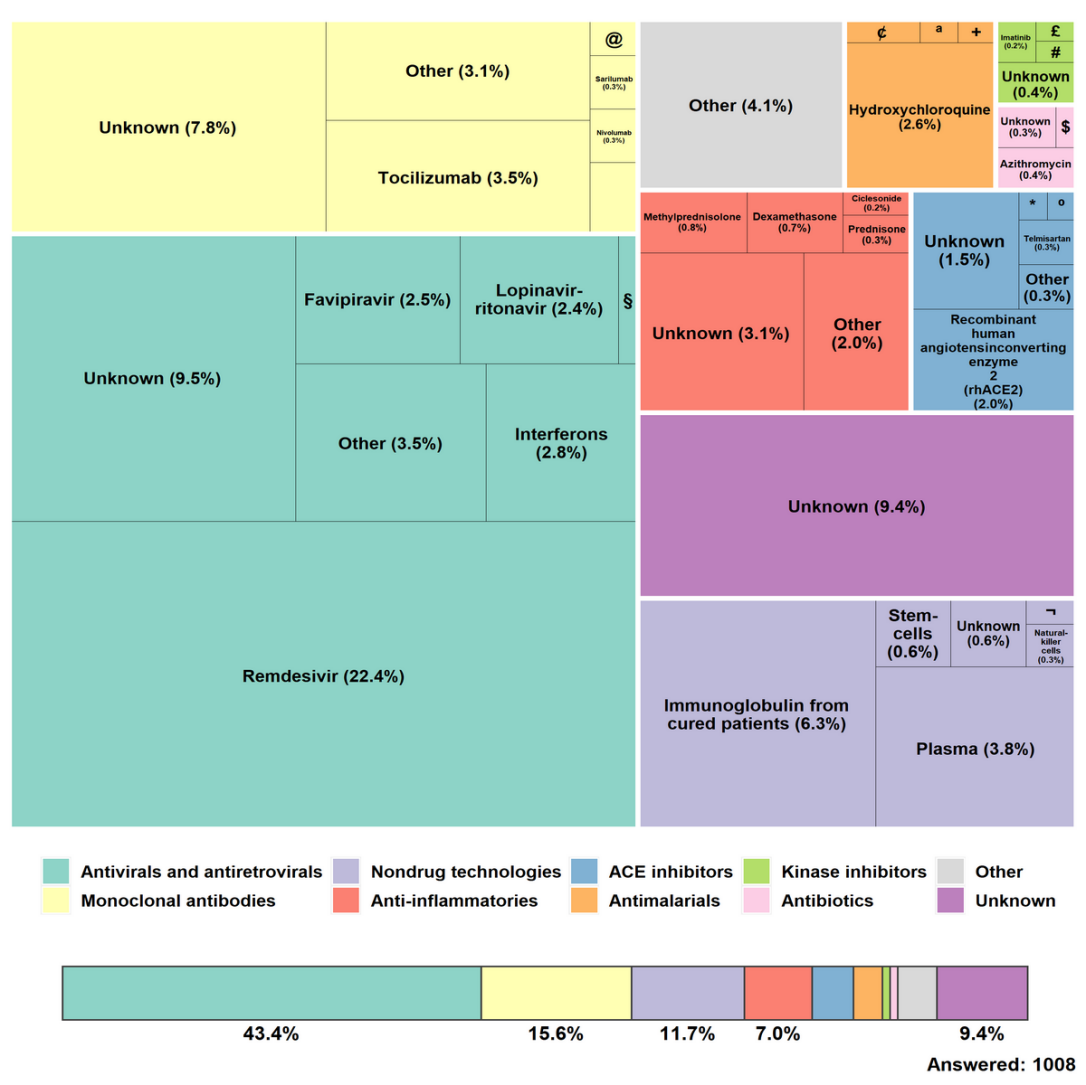
Therapeutics chosen by the respondents to be most likely to succeed in treating COVID-19. § Umifenovir (0.69%); ¢ Unknown antimalarial (0.20%); ª Mefloquine (0.10%); + Chloroquine (0.10%); $ Other antibiotic (0.10%); * Losartan (0.10%); º Captopril (0.10%); @ Bevacizumab (0.20%); £ Acalabrutinib (0.10%); # Other kinase inhibitor (0.10%); ¬ Other nondrug (0.10%).

A great number of therapeutic options for COVID-19 are currently in clinical trials; however, to offer the respondents a short list of options, we included only those that had at least two registered clinical trials by April 23, 2020. We classified these therapeutic options into eight groups of broad treatment types [5] and asked the respondents which group would be the most likely to succeed as a COVID-19 treatment in the next 6 months. The selected group led to a particular question containing several treatment options, of which the respondents were asked to select the most promising. As shown in Figure 4, antivirals and antiretrovirals were believed to be by far the most promising group, selected by 43.4% (437/1008) of the respondents who answered this question. These were followed by monoclonal antibodies (157/1008, 15.6%) and nondrug technologies (118/1008, 11.7%). All the other groups were chosen by fewer than 10.0% of the respondents, with kinase inhibitors and antibiotics being chosen by less than 1% (8/1008, 0.8%). Approximately 4.0% (38/1008) of respondents said they believed there were other broad treatment approaches that were more likely to succeed than the eight listed in the questionnaire, and 9.42% (95/1008) expressed the view that the answer to this question is still unknown. In five of the questions—when the respondents were asked to specify the vaccine, the broad treatment, the antiviral/antiretroviral, the anti-inflammatory, and the nondrug technology—a substantially higher number of respondents with some knowledge of the subject marked “unknown” than those with good knowledge.

Considering all the eight groups together, only 9 of the 36 possible treatment options were chosen by more than 2.5% of the respondents. Remdesivir, from Gilead Sciences, was by far the top choice, being selected by 22.4% (225/1008) of the total respondents. Remdesivir was also the only treatment for which there was any statistically significant difference (*P*=.05) between the responses given by the two groups of respondents (with good knowledge and some degree of knowledge on the subject). The respondents with good knowledge were almost twice as likely to select remdesivir than the ones with some knowledge. Although the group of monoclonal antibodies ranked second, it attracted a high percentage of “unknown” (79/1008, 7.8%) and “other” (31/1008, 3.1%) responses. Of the four monoclonal antibodies in this group, tocilizumab (35/1008, 3.5%) was the preferred option. In terms of individual treatments, the top two choices after remdesivir were both nondrug technologies: immunoglobulin from cured patients (64/1008, 6.3%) and plasma (38/1008, 3.8%). The other treatments selected by at least 2.5% of the respondents are interferons (28/1008, 2.8%), hydroxychloroquine (26/1008, 2.6%), favipiravir (25/1008, 2.5%), lopinavir-ritonavir (24/1008, 2.4%), and recombinant human angiotensin-converting enzyme 2 (rhACE2) (20/1008, 2%).

## Discussion

### Principal Findings

Although experimental therapies for COVID-19 are listed in the WHO’s Research and Development (R&D) Blueprint of COVID-19 experimental treatments [5] and some other recent scientific papers [6], the absence of an effective specific treatment has led to dissent among the stakeholders invested in finding a cure for COVID-19. Diverging views are found beyond medical and biomedical scientists, being found in a variety of health professionals, managers, policy makers, etc. Although this lack of consensus regarding treatment for COVID-19 was also observed among the virus-related researchers who participated in this study, a great number of them (437/1008, 43.4%) stated a preference for antivirals and antiretrovirals over the seven other broad treatment groups addressed in this study.

In the antivirals and antiretrovirals group, remdesivir was the drug that most respondents believed to be the most promising for treating patients with COVID-19. Remdesivir is a nucleoside analog drug with extensive antiviral activity, and it is effective in the treatment of the lethal Ebola and Nipah viruses [13]. Its use in patients with COVID-19 started after a high-profile publication reported on its compassionate use in a 53-patient case series [14]. This publication showed clinical improvement in 36 out of 53 patients (68%); however, it was not randomized, so other evidence was needed [6]. Another published study reported on the use of remdesivir in 236 patients in 10 hospitals in Wuhan, China, in a double-blind, placebo-controlled, multicenter randomized trial. Remdesivir was not associated with a difference in the primary outcome of time to clinical improvement. This study was stopped early because no further patients meeting the eligibility criteria were admitted to hospital in Wuhan after some weeks [15].

On April 23, 2020, five clinical trials evaluating the use of remdesivir in patients with COVID-19 were registered at ClinicalTrials.gov. Four months later, this number had increased to >40. The most recent systematic review and network meta-analysis of drug treatments for COVID-19 shows, with moderate certainty, the benefits of the use of remdesivir both for the resolution of symptoms and the duration of mechanical ventilation. However, there are still uncertainties about its effect on mortality and other important outcomes [16].

Additionally in the group of antivirals and antiretrovirals, the respondents considered interferons, favipiravir, and lopinavir-ritonavir to be promising treatments for COVID-19. Interferons are often evaluated as candidates for the treatment of emerging viral infections before specific treatments are developed because of their unspecific antiviral effects [17]. They are usually prescribed in combination with other drugs, and this was indeed the case in most of the clinical trials involving interferons that were registered by April 23, 2020. Several types of interferons are available for antiviral treatment of patients with COVID-19, including alpha [16], beta [18], and lambda [19] interferons. As for favipiravir, limited clinical experience has been reported to date [6]; however, recent experimental treatments with favipiravir in combination with interferon alfa reported significant improvements in chest imaging [20]. In the case of lopinavir-ritonavir, a recent evaluation showed no observed benefit in 99 hospitalized adult patients [21].

Nondrugs are also considered to be promising for the treatment of patients with COVID-19. Immunotherapy with IgG can increase immune response in newly infected patients, reducing the disease burden [22]. Plasma collected from recovered patients functions similarly. In a recent experiment, 5 severely ill patients in China showed improvement after receiving convalescent plasma [23]. However, despite its potential usefulness, the absence of large-scale trials and more rigorous investigations of convalescent plasma is an obstacle to its more widespread use in patients with COVID-19 [24].

Another potential drug available to treat COVID-19 is a member of the monoclonal antibody group. Tocilizumab is a humanized monoclonal antibody against the interleukin-6 receptor, which has been reported to be one of the most important cytokines involved in COVID-19 [25]. A recent publication showed clinical improvement of respiratory function in 91% of patients who were prescribed tocilizumab [26]. However, in the absence of other studies, its use should be approached with caution [6].

On April 23, 2020, hydroxychloroquine was the drug being tested in the highest number of clinical trials. It was considered to be a promising treatment for COVID-19 by 2.6% of the respondents. Hydroxychloroquine is commonly used as a treatment for malaria, systemic lupus erythematosus, and rheumatoid arthritis [6]. Despite initial reports of its success [27], recent scientific publications have not shown any significant reduction in mortality, and some have expressed caution because of its potentially harmful effects [28,29].

In the angiotensin-converting enzyme (ACE) inhibitors group, rhACE2 was another option selected by at least 2% of the respondents. The rationale for its use comes from the assumption that the SARS-CoV-2 virus uses angiotensin-converting enzyme 2 (ACE2) receptors to enter host cells [30]. Hence, exogenously supplementing rhACE2 may block the interaction of the virus with its cellular receptor [31]. However, further clinical studies are needed [30].

Although it was selected only by 0.7% of respondents, dexamethasone is the drug that has probably showed the greatest efficacy in reducing the COVID-19 burden, especially in critically ill patients. After the UK-based Randomised Evaluation of COVID-19 Therapy (RECOVERY) trial reported benefits of the use of dexamethasone [32], other trials and a recent meta-analysis showed that the use of dexamethasone and other systemic corticosteroids is associated with a 28-day reduction in patient mortality [33].

Regardless of the importance of pharmacological treatment to reduce the short- and medium-term burden of COVID-19, there is a consensus in the scientific community that large-scale vaccination will be the most effective long-term strategy to end the pandemic [7,34]. It usually takes years to develop a new vaccine; however, the need for rapid development has required a completely different paradigm [8]. Yet, even considering the amount of progress made in the few months after our survey, in September 2020, most vaccine candidates were still in the preclinical phase, and dozens were in phase 1 or 2. Some vaccines were in phase 3. This is the case, for example, for the vaccine candidates from BioNTech/Pfizer and from the Gamaleya Research Institute, which showed good results in terms of safety and immunogenicity [35,36]. In December 2020, the BioNTech/Pfizer vaccine was the first to be made available worldwide after being approved by regulatory bodies in the United States, Europe, Canada, and the United Kingdom. All these candidates highlight the diversity of vaccine development technology platforms, including recent advances not yet used in any licensed viral vaccine, such as RNA- and DNA-based technologies [7].

Considered the most promising vaccine candidate by the respondents of this survey, ChAdOx1, from the University of Oxford, uses a more traditional nonreplicating viral vector technological platform. This type of platform offers long-term stability, induces a strong immune response, and offers high levels of protein expression. Because some vaccines are already using this platform for other diseases, there is also the advantage of potentially using the existing industrial capacity for its production [7]. A preliminary report of a phase 1/2 single-blind randomized controlled trial of ChAdOx1 showed an acceptable safety profile and homologous increase in antibody responses [37]. In January 2020, this vaccine was also approved for wide use in the United Kingdom and Mexico.

A nonreplicating viral vector platform is also being used for the Ad5-nCoV vaccine candidate, from CanSino Biologics, which was the third most highly ranked vaccine candidate in the study. Ad5-nCoV has already been indicated to be tolerable and immunogenic at 28 days postvaccination [38], and it is currently being used in the Chinese military [39]. The second most selected vaccine, mRNA-1273, developed by Moderna, uses innovative messenger RNA (mRNA) technology and was also approved for emergency use in Israel, the United States, and Canada. Because this virus uses synthetic processes and does not require culture or fermentation, it may be easier for Moderna to produce a large number of doses with its RNA-based technology [8,34].

### Final Remarks

This study presents the results of a global survey of over 1000 virus specialists with knowledge on the prevention and treatment of coronavirus-related human diseases that took place in May 2020. A great number of the respondents believed that antiviral and antiretroviral therapies were the most likely treatments to succeed in the fight against COVID-19. Of the 36 options, grouped into eight broad treatment types, remdesivir and immunoglobulin from cured patients were regarded as the most likely to prove effective. Although remdesivir is currently being used in clinical practice with guidelines [40], the respondents did not expect that dexamethasone and other systemic steroids could have an important role in treatment [33].

As mentioned, the race for a COVID-19 vaccine has given rise to a large number of other vaccine candidates, with some already showing good results in terms of safety and immunogenicity and others already being used in a few countries. Of the vaccine candidates presented to the respondents, ChAdOx1 and mRNA-1273 were considered the two most promising options for COVID-19 prevention. Also, most respondents indicated that it was likely that a vaccine would be available within 18 months. This seemed very optimistic, given that a new vaccine usually takes 10 years to develop. Even the fast-tracked development of the first Ebola vaccine required 5 years [7]. However, changes in the clinical trial process were implemented worldwide in an attempt to accelerate the discovery of an entirely new vaccine to prevent COVID-19 [8]. These changes were already taking place in May 2020; this may help us understand why the respondents felt it would take such a short time to develop a vaccine, which is exactly what happened a few months later.

Due to the tremendous scientific efforts to end the COVID-19 pandemic, much has changed since we conducted this survey in May 2020. As a large amount of new information on COVID-19 is published daily, the issues addressed in this study are constantly evolving; therefore, the current state of knowledge is different from that in May 2020. Yet, comparing what was known back at the beginning of the fight against COVID-19 with what is known today, many of the results reported in this study are in line with what is currently happening. Thus, if the future confirms the expectations of the researchers who participated in this study, the discovery that will bring an end to the COVID-19 pandemic will not be very far away.

## Appendix

Multimedia Appendix 1 Clinical trials related to COVID-19.

Multimedia Appendix 2 Statistics for the responses to the survey.

## Abbreviations

AACT: Aggregate Analysis of ClinicalTrials.gov
ACE: angiotensin-converting enzyme
ACE2: angiotensin-converting enzyme 2
CSV: comma-separated values
MeSH: Medical Subject Headings
R&D: research and development
RECOVERY: Randomised Evaluation of COVID-19 Therapy
SARS-CoV: severe acute respiratory syndrome coronavirus
SCI-EXPANDED: Science Citation Index Expanded
WHO: World Health Organization
WoS: Web of Science
