# Supplementary material for: Expert Opinions on the Most Promising Treatments and Vaccine Candidates for COVID-19: Global Cross-sectional Survey of Virus Researchers in the Early Months of the Pandemic
Source: JMIR Public Health Surveill. 2021 Feb 26;7(2):e22483. doi: 10.2196/22483 (PMC7919842; doi:10.2196/22483)
Supplement: Multimedia Appendix 1 [file publichealth_v7i2e22483_app1.docx]

**Supplementary Material 1**

# Mapping of clinical trials

# Methods

We mapped the clinical trials related to COVID-19 registered at ClinicalTrials.gov using the Aggregate Analysis of ClinicalTrials.gov (AACT) database, maintained by the Clinical Trials Transformation Initiative (<https://aact.ctti-clinicaltrials.org/>). The search was carried out on April, 23, 2020, via the PostgreSQL AACT connection. It retrieved 406 clinical trials with coronavirus as the variable condition (diseases being tested). The records were imported to VantagePoint 11.0, where we selected only the trials related to COVID-19 by searching the fields condition, title, outcome measure, keywords, and Medical Subject Headings (MeSH) conditions for the following descriptors: “Covid-19,” Covid19, “Covid 19,” “Coronavirus-19,” “Coronavirus 19,” “Coronavirus 2019,” “Sars-CoV2,” “SARS-CoV-2,” “SARS COV 2,” “Novel Coronavirus,” “2019 Novel Coronavirus,” “2019-Novel coronavirus,” “2019-nCoV,” 2019nCoV, and “Coronavirus Disease 2019”.

This search retrieved 361 clinical trials: 237 interventional studies, where biomedical interventions are assigned and tested, and 124 observational studies, where there is no specific intervention assigned by the principal investigator. Considering the purpose of this study, observational studies were not included in the analysis. Of the 237 interventional studies, only the ones for drugs (173) and biologicals/vaccines (31) were selected. Clinical trials whose status was withdrawn (2) or suspended (1) were excluded. To select only clinical trials of vaccine candidates, we searched in biologicals/vaccines for the terms “vaccine” and “vaccines” in the following fields: interventions, keywords, MeSH interventions, outcome measures, study designs, and title. At the end of these procedures, 170 clinical trials with drug interventions and six clinical trials with vaccine interventions were selected for analysis.

MeSH is the U.S. National Library of Medicine controlled and hierarchically-organized vocabulary ([ncbi.nlm.nih.gov/mesh](https://www.ncbi.nlm.nih.gov/mesh)) used for indexing articles for PubMed ([pubmed.ncbi.nlm.nih.gov/](https://pubmed.ncbi.nlm.nih.gov/)). Keywords from MeSH are given by the principal investigator when they register the clinical trial, and the AACT classifies these keywords as MeSH conditions (keywords related to diseases) and MeSH interventions (keywords related to chosen interventions). There are co-occurrences among and within these keywords, which can be used to produce networks. For drug clinical trials, we built a network of countries and a two-mode network of MeSH conditions and MeSH interventions. It was not possible to build any networks of vaccine clinical trials due to the absence of co-occurrences. All the networks were built in Gephi 0.9.2 from co-occurrence matrices produced in VantagePoint 11.0. The Force Atlas 2 algorithm provided the networks’ layout, and the size of the nodes was given by their weighted degree (sum of connected nodes weighted by the nodes’ co-occurrence). Betweenness centrality and Eigenvector centrality were also used to describe the networks. The former calculates how many times a node appears on the shortest paths connecting nodes in the network, and the latter measures how important a node is in a network based on its direct and indirect connections.

# Results

Figure 1. shows an overview of the clinical trials related to COVID-19 included in ClinicalTrials.gov until April 23, 2020. Five of the six clinical trials for vaccines were both recruiting and in phase 1. Three are expected to finish in 2021, one in 2022, and two in 2024. They are being conducted in China (4), the United States (1), and the United Kingdom (1). As for the drug trials, 46.5% were recruiting, 44.7% were not yet recruiting, and only 1% were completed on April 23, 2020. Most of the drug trials were in phase 2 (48.8%) or phase 3 (25.9%). About 60% of them are expected to finish in 2020, and 26.5% in 2021. Although there are clinical trials for drugs in progress in 35 countries, most are in the United States (29.4%), China (11.2%), France (7.6%), and Spain (7.1%).

Many of the drug trials are collaborations between institutions from different countries, which are being conducted at more than one facility at the same time. These collaborations are represented in the country network (Figure 1. ). The nodes represent the countries and the lines are collaborations between them. Taking into account not only the number of trials but also the number of collaborations (Eigenvector centrality), the United States is the most central node in the network, followed by the United Kingdom, France, and Italy.

Figure 1. also shows the two-mode network of MeSH conditions and MeSH interventions. Desaturated blue nodes refer to the top ten MeSH conditions, visible on the left-hand side of the network. All the other nodes represent MeSH interventions (that occur more than once), which are colored according to the broad treatment types they belong to. Desaturated blue lines represent co-occurrences between conditions (a clinical trial may use the same protocol for different diseases), green lines represent co-occurrences between interventions (drugs can be tested on a combination of two or more conditions), and red lines show co-occurrences between conditions and interventions (only co-occurrences with weight above ten are depicted).

The most significant node among the MeSH conditions is “coronavirus infections,” followed by “SARS.” About 73% of the clinical trials that have “coronavirus infections” as a MeSH condition co-occur with “SARS,” making this the highest co-occurrence between MeSH conditions. For MeSH interventions, “hydroxychloroquine” has the highest weight. Its use is reported in 27.6% of all the drug trials, 12.7% in combination with ritonavir and lopinavir, and 10.6% in combination with azithromycin. The nodes “Ritonavir” and “Lopinavir” are the most central in terms of Eigenvector centrality. Some nodes represent not only one drug, but a group of drugs (e.g., “antiviral agents” includes clinical trials of remdesivir, and “monoclonal antibodies” contains lenzilumab). In the broad treatments groups, the highest co-occurrences are between drugs in the same group. Associations of two antivirals/antiretrovirals are the most common, followed by associations of two anti-inflammatories. As for associations of two different broad treatments, the most frequent is antivirals/antiretrovirals in association with anti-inflammatories, followed by antimalarials in association with antibiotics.

Analyzing the most important co-occurrences between conditions and interventions, hydroxychloroquine has the highest betweenness centrality and is the only drug being tested for all the conditions in the network. Meanwhile, “coronavirus infections” came out with the highest Eigenvector centrality: not only is it connected to all the conditions (being tested in the same trial) but also to almost all the interventions. The COVID-19 clinical trials with lopinavir and ritonavir are also for SARS, pneumonia, and infection.


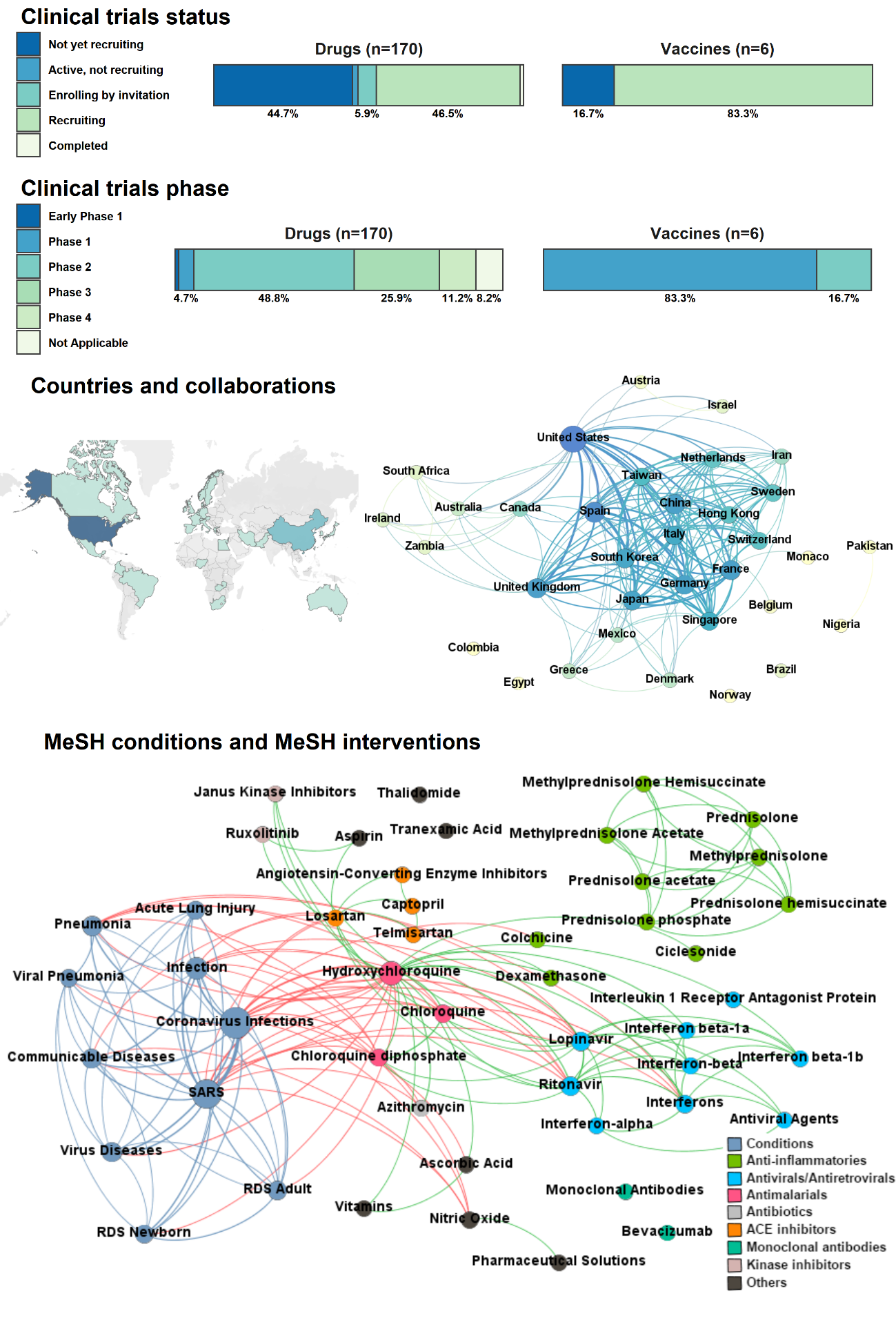


**Figure 1. COVID-19 clinical trials overview**
