# Supplementary material for: Expert Opinions on the Most Promising Treatments and Vaccine Candidates for COVID-19: Global Cross-sectional Survey of Virus Researchers in the Early Months of the Pandemic
Source: JMIR Public Health Surveill. 2021 Feb 26;7(2):e22483. doi: 10.2196/22483 (PMC7919842; doi:10.2196/22483)
Supplement: Multimedia Appendix 2 [file publichealth_v7i2e22483_app2.pdf]

Please indicate your knowledge level on prevention and treatment of coronavirus-related human diseases

| main_question | n | % |
|---|---|---|
| I have good knowledge | 735 | 57.6% |
| I have some knowledge | 495 | 38.8% |
| I have no knowledge | 45 | 3.5% |
| Total | 1275 | |

Is it likely that a vaccine for COVID-19 will be available in the next 18 months?

| knowledge | total_knowledge | answer_vac1 | n | % |
|---|---|---|---|---|
| Good knowledge | 729 | Likely | 457 | 37.5% |
| Good knowledge | 729 | Unlikely | 167 | 13.7% |
| Good knowledge | 729 | Unknown | 105 | 8.6% |
| Some knowledge | 490 | Likely | 309 | 25.3% |
| Some knowledge | 490 | Unlikely | 122 | 10.0% |
| Some knowledge | 490 | Unknown | 59 | 4.8% |
| Total | 1219 | | | |

In the next 18 months, of all vaccine candidates currently in phase 1 or 2 clinical trials, which of these do you consider most likely to succeed in preventing COVID-19? + Which of all vaccine candidates currently in phase 1 or 2 clinical trials do you consider most likely to succeed in preventing COVID-19?

| knowledge | Total | answer_vac1 | answer_vac2 | n | % |
|---|---|---|---|---|---|
| Good knowledge | 435 | Likely | Pathogen-specific aAPC from Shenzhen Geno-Immune Medical Institute | 16 | 1.6% |
| Good knowledge | 435 | Likely | Ad5-nCoV from CanSino Biologicals | 26 | 2.6% |
| Good knowledge | 435 | Likely | INO-4800 from Inovio Pharmaceuticals | 8 | 0.8% |
| Good knowledge | 435 | Likely | LV-SMENP-DC from Shenzhen Geno-Immune Medical Institute | 5 | 0.5% |
| Good knowledge | 435 | Likely | mRNA-1273 from Moderna | 39 | 3.9% |
| Good knowledge | 435 | Likely | ChAdOx1 from University of Oxford | 100 | 9.9% |
| Good knowledge | 435 | Likely | Other | 31 | 3.1% |
| Good knowledge | 435 | Likely | Unknown | 105 | 10.4% |
| Good knowledge | 435 | Likely | I prefer not to answer | 105 | 10.4% |
| Good knowledge | 160 | Unlikely | Pathogen-specific aAPC from Shenzhen Geno-Immune Medical Institute | 1 | 0.1% |
| Good knowledge | 160 | Unlikely | Ad5-nCoV from CanSino Biologicals | 3 | 0.3% |
| Good knowledge | 160 | Unlikely | INO-4800 from Inovio Pharmaceuticals | 1 | 0.1% |
| Good knowledge | 160 | Unlikely | LV-SMENP-DC from Shenzhen Geno-Immune Medical Institute | 2 | 0.2% |
| Good knowledge | 160 | Unlikely | mRNA-1273 from Moderna | 14 | 1.4% |
| Good knowledge | 160 | Unlikely | ChAdOx1 from University of Oxford | 35 | 3.5% |
| Good knowledge | 160 | Unlikely | Other | 6 | 0.6% |
| Good knowledge | 160 | Unlikely | Unknown | 55 | 5.5% |
| Good knowledge | 160 | Unlikely | I prefer not to answer | 43 | 4.3% |
| Some knowledge | 296 | Likely | Pathogen-specific aAPC from Shenzhen Geno-Immune Medical Institute | 10 | 1.0% |
| Some knowledge | 296 | Likely | Ad5-nCoV from CanSino Biologicals | 7 | 0.7% |
| Some knowledge | 296 | Likely | INO-4800 from Inovio Pharmaceuticals | 4 | 0.4% |
| Some knowledge | 296 | Likely | LV-SMENP-DC from Shenzhen Geno-Immune Medical Institute | 6 | 0.6% |
| Some knowledge | 296 | Likely | mRNA-1273 from Moderna | 16 | 1.6% |
| Some knowledge | 296 | Likely | ChAdOx1 from University of Oxford | 45 | 4.5% |
| Some knowledge | 296 | Likely | Other | 19 | 1.9% |
| Some knowledge | 296 | Likely | Unknown | 114 | 11.3% |
| Some knowledge | 296 | Likely | I prefer not to answer | 75 | 7.4% |
| Some knowledge | 117 | Unlikely | Pathogen-specific aAPC from Shenzhen Geno-Immune Medical Institute | 0 | 0.0% |
| Some knowledge | 117 | Unlikely | Ad5-nCoV from CanSino Biologicals | 3 | 0.3% |
| Some knowledge | 117 | Unlikely | INO-4800 from Inovio Pharmaceuticals | 0 | 0.0% |
| Some knowledge | 117 | Unlikely | LV-SMENP-DC from Shenzhen Geno-Immune Medical Institute | 0 | 0.0% |
| Some knowledge | 117 | Unlikely | mRNA-1273 from Moderna | 5 | 0.5% |
| Some knowledge | 117 | Unlikely | ChAdOx1 from University of Oxford | 12 | 1.2% |
| Some knowledge | 117 | Unlikely | Other | 7 | 0.7% |
| Some knowledge | 117 | Unlikely | Unknown | 57 | 5.7% |
| Some knowledge | 117 | Unlikely | I prefer not to answer | 33 | 3.3% |
| Total | 1008 | | | | |

In the next six months, which one of these broad approaches do you consider most likely to succeed as COVID-19 treatment?

| knowledge | total_knowledge | answer_drug1 | n | % |
|---|---|---|---|---|
| Good knowledge | 694 | Antivirals and antiretrovirals | 311 | 26.8% |
| Good knowledge | 694 | Antimalarials | 17 | 1.5% |
| Good knowledge | 694 | Antibiotics | 5 | 0.4% |
| Good knowledge | 694 | Angiotensin-converting-enzyme (ACE) inhibitors | 23 | 2.0% |
| Good knowledge | 694 | Anti-inflammatories | 40 | 3.5% |
| Good knowledge | 694 | Non-drug technologies (e.g. plasma, stem-cells) | 83 | 7.2% |
| Good knowledge | 694 | Monoclonal antibodies | 114 | 9.8% |
| Good knowledge | 694 | Kinase inhibitors | 2 | 0.2% |
| Good knowledge | 694 | Other | 24 | 2.1% |
| Good knowledge | 694 | Unknown | 47 | 4.1% |
| Good knowledge | 694 | I prefer not to answer | 28 | 2.4% |
| Some knowledge | 465 | Antivirals and antiretrovirals | 168 | 14.5% |
| Some knowledge | 465 | Antimalarials | 15 | 1.3% |
| Some knowledge | 465 | Antibiotics | 3 | 0.3% |
| Some knowledge | 465 | Angiotensin-converting-enzyme (ACE) inhibitors | 26 | 2.2% |
| Some knowledge | 465 | Anti-inflammatories | 46 | 4.0% |
| Some knowledge | 465 | Non-drug technologies (e.g. plasma, stem-cells) | 35 | 3.0% |
| Some knowledge | 465 | Monoclonal antibodies | 86 | 7.4% |
| Some knowledge | 465 | Kinase inhibitors | 6 | 0.5% |
| Some knowledge | 465 | Other | 17 | 1.5% |
| Some knowledge | 465 | Unknown | 48 | 4.1% |
| Some knowledge | 465 | I prefer not to answer | 15 | 1.3% |
| Total | 1159 | | | |

In the next six months, of all *drugs* with at least two registred clinical trials, which one of these do you consider most likely to succeed as COVID-19 treatment?

| knowledge | Total | answer_vac1 | answer_vac2 | n | % |
|---|---|---|---|---|---|
| Good knowledge | 305 | Antivirals and antiretrovirals | Favipiravir | 13 | 1.3% |
| Good knowledge | 305 | Antivirals and antiretrovirals | I prefer not to answer | 21 | 2.2% |
| Good knowledge | 305 | Antivirals and antiretrovirals | Interferons | 18 | 1.9% |
| Good knowledge | 305 | Antivirals and antiretrovirals | Lopinavir-ritonavir | 17 | 1.8% |
| Good knowledge | 305 | Antivirals and antiretrovirals | Other | 26 | 2.7% |
| Good knowledge | 305 | Antivirals and antiretrovirals | Remdesivir | 162 | 16.8% |
| Good knowledge | 305 | Antivirals and antiretrovirals | Umifenovir | 3 | 0.3% |
| Good knowledge | 305 | Antivirals and antiretrovirals | Unknown | 45 | 4.7% |
| Some knowledge | 166 | Antivirals and antiretrovirals | Favipiravir | 12 | 1.2% |
| Some knowledge | 166 | Antivirals and antiretrovirals | I prefer not to answer | 13 | 1.3% |
| Some knowledge | 166 | Antivirals and antiretrovirals | Interferons | 10 | 1.0% |
| Some knowledge | 166 | Antivirals and antiretrovirals | Lopinavir-ritonavir | 7 | 0.7% |
| Some knowledge | 166 | Antivirals and antiretrovirals | Other | 9 | 0.9% |
| Some knowledge | 166 | Antivirals and antiretrovirals | Remdesivir | 64 | 6.6% |
| Some knowledge | 166 | Antivirals and antiretrovirals | Umifenovir | 0 | 0.0% |
| Some knowledge | 166 | Antivirals and antiretrovirals | Unknown | 51 | 5.3% |
| Good knowledge | 17 | Antimalarials | Hydroxychloroquine | 14 | 1.4% |
| Good knowledge | 17 | Antimalarials | I prefer not to answer | 1 | 0.1% |
| Good knowledge | 17 | Antimalarials | Mefloquine | 1 | 0.1% |
| Good knowledge | 17 | Antimalarials | Unknown | 1 | 0.1% |
| Some knowledge | 15 | Antimalarials | Chloroquine | 1 | 0.1% |
| Some knowledge | 15 | Antimalarials | Hydroxychloroquine | 12 | 1.2% |
| Some knowledge | 15 | Antimalarials | I prefer not to answer | 1 | 0.1% |
| Some knowledge | 15 | Antimalarials | Unknown | 1 | 0.1% |
| Good knowledge | 5 | Antibiotics | Azithromycin | 3 | 0.3% |
| Good knowledge | 5 | Antibiotics | Other | 1 | 0.1% |
| Good knowledge | 5 | Antibiotics | Unknown | 1 | 0.1% |
| Some knowledge | 3 | Antibiotics | Azithromycin | 1 | 0.1% |
| Some knowledge | 3 | Antibiotics | Other | 0 | 0.0% |
| Some knowledge | 3 | Antibiotics | Unknown | 2 | 0.2% |
| Good knowledge | 23 | Angiotensin-converting-enzyme (ACE) inhibitors | Captopril | 1 | 0.1% |
| Good knowledge | 23 | Angiotensin-converting-enzyme (ACE) inhibitors | I prefer not to answer | 4 | 0.4% |
| Good knowledge | 23 | Angiotensin-converting-enzyme (ACE) inhibitors | Other | 1 | 0.1% |
| Good knowledge | 23 | Angiotensin-converting-enzyme (ACE) inhibitors | Recombinant human angiotensinconverting enzyme 2 | 11 | 1.1% |
| Good knowledge | 23 | Angiotensin-converting-enzyme (ACE) inhibitors | Telmisartan | 0 | 0.0% |

| | | | | | |
|---|---|---|---|---|---|
| Good knowledge | 23 | Angiotensin-converting-enzyme (ACE) inhibitors | Unknown | 6 | 0.6% |
| Some knowledge | 26 | Angiotensin-converting-enzyme (ACE) inhibitors | I prefer not to answer | 2 | 0.2% |
| Some knowledge | 26 | Angiotensin-converting-enzyme (ACE) inhibitors | Losartan | 1 | 0.1% |
| Some knowledge | 26 | Angiotensin-converting-enzyme (ACE) inhibitors | Other | 2 | 0.2% |
| Some knowledge | 26 | Angiotensin-converting-enzyme (ACE) inhibitors | Recombinant human angiotensinconverting enzyme 2 | 9 | 0.9% |
| Some knowledge | 26 | Angiotensin-converting-enzyme (ACE) inhibitors | Telmisartan | 3 | 0.3% |
| Some knowledge | 26 | Angiotensin-converting-enzyme (ACE) inhibitors | Unknown | 9 | 0.9% |
| Good knowledge | 39 | Anti-inflammatories | Ciclesonide | 0 | 0.0% |
| Good knowledge | 39 | Anti-inflammatories | Dexamethasone | 3 | 0.3% |
| Good knowledge | 39 | Anti-inflammatories | I prefer not to answer | 7 | 0.7% |
| Good knowledge | 39 | Anti-inflammatories | Methylprednisolone | 4 | 0.4% |
| Good knowledge | 39 | Anti-inflammatories | Other | 14 | 1.4% |
| Good knowledge | 39 | Anti-inflammatories | Prednisone | 2 | 0.2% |
| Good knowledge | 39 | Anti-inflammatories | Unknown | 9 | 0.9% |
| Some knowledge | 45 | Anti-inflammatories | Ciclesonide | 2 | 0.2% |
| Some knowledge | 45 | Anti-inflammatories | Dexamethasone | 4 | 0.4% |
| Some knowledge | 45 | Anti-inflammatories | I prefer not to answer | 6 | 0.6% |
| Some knowledge | 45 | Anti-inflammatories | Methylprednisolone | 4 | 0.4% |
| Some knowledge | 45 | Anti-inflammatories | Other | 6 | 0.6% |
| Some knowledge | 45 | Anti-inflammatories | Prednisone | 1 | 0.1% |
| Some knowledge | 45 | Anti-inflammatories | Unknown | 22 | 2.3% |
| Good knowledge | 112 | Monoclonal antibodies | Bevacizumab | 0 | 0.0% |
| Good knowledge | 112 | Monoclonal antibodies | I prefer not to answer | 24 | 2.5% |
| Good knowledge | 112 | Monoclonal antibodies | Leronlimab | 2 | 0.2% |
| Good knowledge | 112 | Monoclonal antibodies | Nivolumab | 2 | 0.2% |
| Good knowledge | 112 | Monoclonal antibodies | Other | 20 | 2.1% |
| Good knowledge | 112 | Monoclonal antibodies | Sarilumab | 0 | 0.0% |
| Good knowledge | 112 | Monoclonal antibodies | Tocilizumab | 22 | 2.3% |
| Good knowledge | 112 | Monoclonal antibodies | Unknown | 42 | 4.3% |
| Some knowledge | 84 | Monoclonal antibodies | Bevacizumab | 2 | 0.2% |
| Some knowledge | 84 | Monoclonal antibodies | I prefer not to answer | 15 | 1.6% |
| Some knowledge | 84 | Monoclonal antibodies | Leronlimab | 2 | 0.2% |
| Some knowledge | 84 | Monoclonal antibodies | Nivolumab | 1 | 0.1% |
| Some knowledge | 84 | Monoclonal antibodies | Other | 11 | 1.1% |
| Some knowledge | 84 | Monoclonal antibodies | Sarilumab | 3 | 0.3% |
| Some knowledge | 84 | Monoclonal antibodies | Tocilizumab | 13 | 1.3% |

| | | | | | |
|---|---|---|---|---|---|
| Some knowledge | 84 | Monoclonal antibodies | Unknown | 37 | 3.8% |
| Good knowledge | 2 | Kinase inhibitors | Acalabrutinib | 0 | 0.0% |
| Good knowledge | 2 | Kinase inhibitors | Imatinib | 0 | 0.0% |
| Good knowledge | 2 | Kinase inhibitors | Other | 0 | 0.0% |
| Good knowledge | 2 | Kinase inhibitors | Unknown | 2 | 0.2% |
| Some knowledge | 6 | Kinase inhibitors | Acalabrutinib | 1 | 0.1% |
| Some knowledge | 6 | Kinase inhibitors | Imatinib | 2 | 0.2% |
| Some knowledge | 6 | Kinase inhibitors | Other | 1 | 0.1% |
| Some knowledge | 6 | Kinase inhibitors | Unknown | 2 | 0.2% |
| Good knowledge | 83 | Non-drug technologies (e.g. plasma, stem-cells) | Immunoglobulin from cured patients | 47 | 4.9% |
| Good knowledge | 83 | Non-drug technologies (e.g. plasma, stem-cells) | Natural-killer cells | 2 | 0.2% |
| Good knowledge | 83 | Non-drug technologies (e.g. plasma, stem-cells) | Other | 0 | 0.0% |
| Good knowledge | 83 | Non-drug technologies (e.g. plasma, stem-cells) | Plasma | 27 | 2.8% |
| Good knowledge | 83 | Non-drug technologies (e.g. plasma, stem-cells) | Stem-cells | 5 | 0.5% |
| Good knowledge | 83 | Non-drug technologies (e.g. plasma, stem-cells) | Unknown | 2 | 0.2% |
| Some knowledge | 35 | Non-drug technologies (e.g. plasma, stem-cells) | Immunoglobulin from cured patients | 17 | 1.8% |
| Some knowledge | 35 | Non-drug technologies (e.g. plasma, stem-cells) | Natural-killer cells | 1 | 0.1% |
| Some knowledge | 35 | Non-drug technologies (e.g. plasma, stem-cells) | Other | 1 | 0.1% |
| Some knowledge | 35 | Non-drug technologies (e.g. plasma, stem-cells) | Plasma | 11 | 1.1% |
| Some knowledge | 35 | Non-drug technologies (e.g. plasma, stem-cells) | Stem-cells | 1 | 0.1% |
| Some knowledge | 35 | Non-drug technologies (e.g. plasma, stem-cells) | Unknown | 4 | 0.4% |
| Total | 966 | | | | |

What is the highest degree of education you have completed?

| knowledge | total_knowledge | answer_vac1 | n | % |
|---|---|---|---|---|
| Good knowledge | 626 | Associate's degree | 11 | 1.0% |
| Good knowledge | 626 | Bachelor's degree | 9 | 0.9% |
| Good knowledge | 626 | Doctoral degree | 555 | 52.9% |
| Good knowledge | 626 | Master’s degree | 51 | 4.9% |
| Some knowledge | 424 | Associate's degree | 5 | 0.5% |
| Some knowledge | 424 | Bachelor's degree | 14 | 1.3% |
| Some knowledge | 424 | Doctoral degree | 358 | 34.1% |
| Some knowledge | 424 | Master’s degree | 47 | 4.5% |
| Total | 1050 | | | |

Which occupation type best applies to you?

| knowledge | total_knowledge | answer_vac1 | n | % |
|---|---|---|---|---|
| Good knowledge | 633 | Manager, Executive | 13 | 1.2% |
| Good knowledge | 633 | Masters, PhD Student | 26 | 2.5% |
| Good knowledge | 633 | Other | 27 | 2.5% |
| Good knowledge | 633 | Physician, Clinician | 68 | 6.4% |
| Good knowledge | 633 | Policy maker | 3 | 0.3% |
| Good knowledge | 633 | Professor, Researcher | 450 | 42.5% |
| Good knowledge | 633 | Public Health, Healthcare Professional | 46 | 4.3% |
| Some knowledge | 426 | Manager, Executive | 6 | 0.6% |
| Some knowledge | 426 | Masters, PhD Student | 29 | 2.7% |
| Some knowledge | 426 | Other | 24 | 2.3% |
| Some knowledge | 426 | Physician, Clinician | 20 | 1.9% |
| Some knowledge | 426 | Policy maker | 1 | 0.1% |
| Some knowledge | 426 | Professor, Researcher | 314 | 29.7% |
| Some knowledge | 426 | Public Health, Healthcare Professional | 32 | 3.0% |
| 1059 | | | | |

What type of institution do you work in?

| knowledge | total_knowledge | answer_vac1 | n | % |
|---|---|---|---|---|
| Good knowledge | 628 | Government | 67 | 6.4% |
| Good knowledge | 628 | Hospital or similar organizations | 69 | 6.5% |
| Good knowledge | 628 | Industry | 26 | 2.5% |
| Good knowledge | 628 | University, Research Organization | 466 | 44.2% |
| Some knowledge | 426 | Government | 59 | 5.6% |
| Some knowledge | 426 | Hospital or similar organizations | 35 | 3.3% |
| Some knowledge | 426 | Industry | 18 | 1.7% |
| Some knowledge | 426 | University, Research Organization | 314 | 29.8% |
| Total | 1054 | | | |

How many years of experience you have in your field?

| knowledge | total_knowledge | answer_vac1 | n | % |
|---|---|---|---|---|
| Good knowledge | 634 | Between 10 and 20 years | 195 | 18.4% |
| Good knowledge | 634 | Between 5 and 10 years | 83 | 7.8% |
| Good knowledge | 634 | Less than 5 years | 33 | 3.1% |
| Good knowledge | 634 | More than 20 years | 323 | 30.4% |
| Some knowledge | 427 | Between 10 and 20 years | 139 | 13.1% |
| Some knowledge | 427 | Between 5 and 10 years | 78 | 7.4% |
| Some knowledge | 427 | Less than 5 years | 40 | 3.8% |
| Some knowledge | 427 | More than 20 years | 170 | 16.0% |
| Total | 1061 | | | |

In which region do you live?

| knowledge | total knowledge | answer_vac1 | n | % |
|---|---|---|---|---|
| Good knowledge | 633 | Africa | 43 | 4.1% |
| Good knowledge | 633 | Asia (including Middle East) | 127 | 12.0% |
| Good knowledge | 633 | Australasia or Pacific Islands | 17 | 1.6% |
| Good knowledge | 633 | Europe | 163 | 15.4% |
| Good knowledge | 633 | North America (including Central America and the Caribbean) | 170 | 16.0% |
| Good knowledge | 633 | South America | 113 | 10.7% |
| Some knowledge | 427 | Africa | 33 | 3.1% |
| Some knowledge | 427 | Asia (including Middle East) | 97 | 9.2% |
| Some knowledge | 427 | Australasia or Pacific Islands | 17 | 1.6% |
| Some knowledge | 427 | Europe | 128 | 12.1% |
| Some knowledge | 427 | North America (including Central America and the Caribbean) | 82 | 7.7% |
| Some knowledge | 427 | South America | 70 | 6.6% |
| Total | 1060 | | | |
